# A randomized clinical trial of mindfulness meditation versus exercise in Parkinson’s disease during social unrest

**DOI:** 10.1038/s41531-023-00452-w

**Published:** 2023-01-21

**Authors:** Jojo Yan Yan Kwok, Edmond Pui Hang Choi, Janet Yuen Ha Wong, Kris Yuet Wan Lok, Mu-Hsing Ho, Daniel Yee Tak Fong, Jackie Cheuk Yin Kwan, Shirley Yin Yu Pang, Man Auyeung

**Affiliations:** 1 School of Nursing, Li Ka Shing Faculty of Medicine, The University of Hong Kong, Hong Kong SAR, China; 2 Hong Kong Metropolitan University, Hong Kong SAR, China; 3 The Hong Kong Society for Rehabilitation, Hong Kong SAR, China; 4 Department of Medicine, Queen Mary Hospital, Hong Kong SAR, China; 5 Department of Medicine, Pamela Youde Nethersole Eastern Hospital, Chai Wan, Hong Kong SAR, China

**Keywords:** Parkinson's disease, Human behaviour

## Abstract

Clinical practice guidelines support resilience training and exercise for patients with Parkinson’s disease (PD). This assessor-blinded, randomized clinical trial aimed to compare the effects of a modified mindfulness meditation program versus stretching and resistance training exercise (SRTE) in patients with mild-to-moderate PD. A total of 126 potential participants were enrolled via convenience sampling, of which 68 eligible participants were randomized 1:1 to receive eight weekly 90-min sessions of mindfulness meditation or SRTE. Compared to the SRTE group, generalized estimating equation analyses revealed that the mindfulness group had significantly better improvement in outcomes, particularly for improving depressive symptoms (*d*, −1.66; 95% CI, −3.31 to −0.02) at week 8 and maintaining emotional non-reactivity at week 20 (*d*, 2.08; 95% CI, 0.59 to 3.56). Both groups demonstrated significant immediate, small-moderate effects on cognition (effect size [*d*] = 0.36–0.37, *p* = 0.006–0.011). Compared with the SRTE, mindfulness meditation appeared to be a feasible and promising strategy for managing depressive symptoms and maintaining emotional stability, with comparable benefits on cognitive performance. To combat the psychospiritual and cognitive sequelae of social unrest and COVID-19 pandemic, the integration of mindfulness training into motor-oriented PD rehabilitation protocols is recommended to strengthen the resilience and minimize the psycho-cognitive comorbidities among patients with mild-to-moderate PD.

Trial Registration: HKU Clinical Trials Registry identifier: HKUCTR-2681.

## Introduction

Parkinson’s disease (PD) is the second-most common neurodegenerative disorder worldwide, which severely limits patients’ functional mobility, independence, and health-related quality of life (HRQOL)^1–3^. Pharmacotherapy provides symptomatic relief, however, it is often limited by response fluctuations as the disease progresses. The non-pharmacological, lifestyle approaches including physical activity, stress management, and social support are recommended by clinical guidelines to provide additional symptomatic relief in the illness trajectory^1^. However, maintaining a healthy lifestyle might be difficult in times of adversities such as the 2019–2020 social unrest in Hong Kong and the ongoing coronavirus disease 2019 (COVID-19) pandemic, which are major stressful life events that recently occur^4,5^. Take for example the 2019–2020 Hong Kong social unrest, a series of large-scale demonstrations and protests throughout the city have escalated into conflicting scenes among protestors and police on the streets and other venues such as shopping malls, restaurants, and public transportations^6^. PD patients cannot avoid these scenes when going out or traveling. These unexpected scenes were not only highly stressful, but also exacerbated various motor symptoms including tremors and freezing of gait, which markedly hampered their functional mobility and placed them at high risk of falls and/or injuries. In addition, the different political views among families and friends further shrunken PD patients’ social roles and support network. Although the outbreak of the COVID-19 pandemic in early 2020 largely silenced the protests, the mental health crisis continued due to the fear of infection. Helmich and Bloem^7^ highlighted that successful coping with drastic changes is a dopamine-dependent cognitive process. Owing to the PD-related nigrostriatal dopamine depletion, patients are prone to experience emotional, cognitive, and motor inflexibility, and thus, particularly vulnerable to stressful life events jeopardizing their disease management and prognosis. Hence, stress management and resilience training have emerged as an important topic in contemporary PD care^2^.

Emerging empirical evidence suggests socially engaging activities such as mindfulness and physical exercise can facilitate the psycho-cognitive process of coping and reduce stress^8–10^. A recent survey interviewing 5000 PD patients in the United States found that physical exercise was identified as the most frequently used method (83.1%), while 38.7% of PD respondents who practiced mindfulness noticed improvement in both motor and non-motor symptoms^11^. With the increasing attention of mindfulness in wellness promotion, its research evidence on PD population was just recently evolving. Indeed, mindfulness encompasses a family of practices, including meditation and yoga which are commonly used in healthcare. A clinical trial of yoga involving 138 PD patients demonstrated its significant superior therapeutic effects on alleviating anxiety and depression compared to active exercise control (stretching and resistance training exercises), with similar physical benefits^12^. However, some may argue such positive effects may be contributed by the physical pathways of yoga exercise. Meanwhile, a 2017 systematic review concluded the effects of mindfulness meditation remained inconclusive in PD patients^13^. Hence, this proposed study aimed to further examine the effects of the relatively static, mentally focused mindfulness practice – mindfulness meditation – versus conventional physical exercise on psychological distress (primary outcomes: anxiety and depression), and cognition, mindfulness, physical health, and HRQOL (secondary outcomes) among patients with mild-to-moderate PD, compared to stretching and resistance training exercise. This study provided valuable evidence examining these behavioral approaches and their effects in protecting PD patients against stressors owing to major social, political, and public adversities during the 2019–2020 social unrest in Hong Kong. We hypothesized that patients with PD randomly assigned to receive mindfulness meditation would show a greater improvement in psychological distress and the listed health outcomes than patients receiving physical exercises.

## Results

**Fig. 1:**
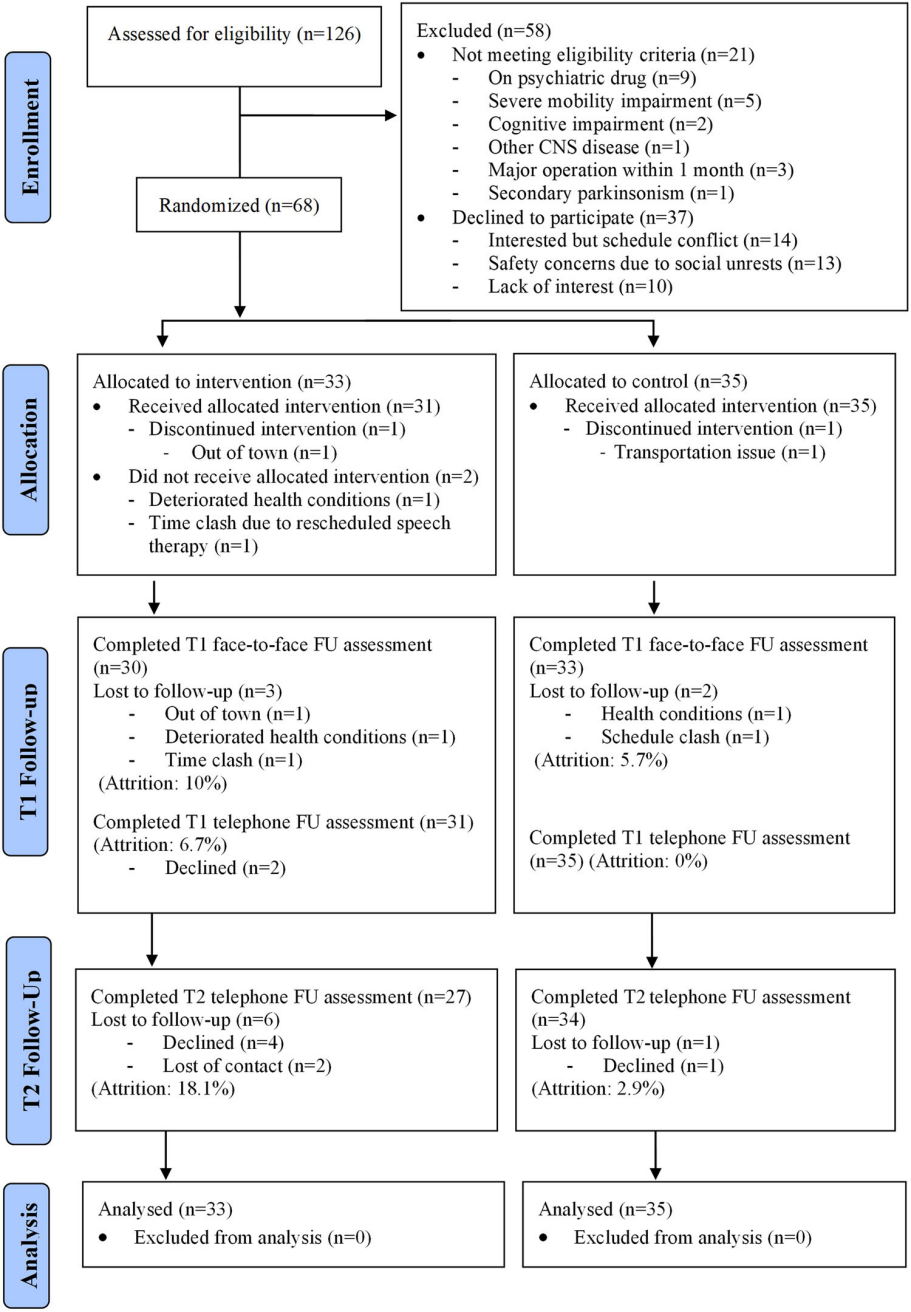
CONSORT 2010 flow diagram. A flow diagram showing the enrolment, allocation, follow-up and analysis of subjects involved in the RCT.

Of 126 potential participants screened, 21 did not meet eligibility criteria and 37 declined to participate because of schedule conflicts (*n* = 14) (Fig. 1). Transportation and safety concerns are also related to the recent outbreak of social movement (*n* = 13) and lack of interest (n = 10) (enrollment rate: 54.0%) (Fig. 1). Of the 68 randomized participants, 33 were in the experimental group (MM) and 35 were in the active control group (SRTE). Two participants randomized to the mindfulness group did not attend any sessions, whereas participants randomized to the SRTE group attended at least two sessions. The mean (SD) attendance rates were 6.03 (1.9) for the mindfulness group and 7.14 (1.29) sessions for the SRTE group; 24 out of 33 participants (72.7%) attended at least six sessions of mindfulness meditation, and 31 out of 35 participants (88.6%) attended at least six sessions of SRTE.

In view of the safety concerns related to ongoing protests during the intervention period (Oct 2019), our team has adopted additional strategies to maintain the safety for participants while accessing to the community centers, including (i) real-time monitoring of the protests situations, and a summary of affected areas/disturbed transportation were sent to the participants via WhatsApp 1.5 h before the intervention sessions, (ii) if there were any protests nearby the community centers, we would reschedule the intervention sessions and make the announcement at least 1.5 h before the intervention sessions, and (iii) all participants were reassured that their safety was the top priority and they could decide to join/skip any sessions without any consequences. Owing to the sudden onset of protests and affected MTR transportation services in areas nearby the community centers, we had to reschedule about 10% of the intervention sessions to other timeslots. For the rescheduled sessions, a lower attendance has been recorded compared to the prescheduled sessions (56% vs 88%). The crude attendance rates were 80.2% for the mindfulness group and 89.3% for the SRTE group. Excluding rescheduled sessions, the attendance rates were 85.3% for the mindfulness group and 89.7% sessions for the SRTE group.

**Fig. 2:**
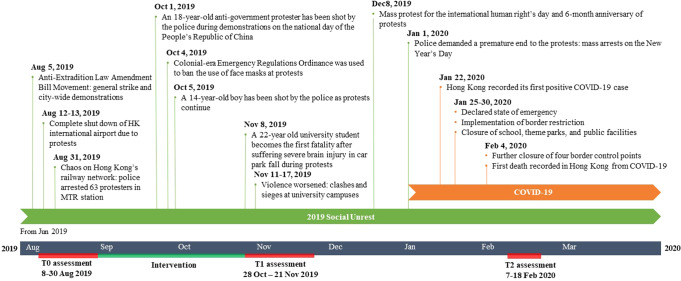
Major events in Hong Kong during the study implementation from Aug, 2019 to Feb, 2020.

Figure 2 shows the timeline of major adverse events in Hong Kong during the study was implemented. 68 participants were recruited and randomized for the first batch of interventions. All self-reported and clinical outcomes were administered in Aug 2019 (T0: baseline) and Nov 2019 (T1: immediate post-intervention). Given the local enforcement of stringent public health measures toward the COVID-19 pandemic since Jan 2020 (including the suspension of all research and non-clinical services), we were not able to conduct any face-to-face T2 assessment in Feb 2020. Only self-reported outcomes were collected via telephone calls at T2. The overall drop-out rates were 5 of 68 (7.4%) at T1 (MM: 10%; SRTE: 5.7%) and 7 of 68 (10.3%) at T2 (MM: 18.1%; SRTE: 2.9%). With the mandatory closing of all community-based leisure and recreational facilities and suspending research and non-clinical services since Jan 2020, all recruitment and community group-based rehabilitation services were suspended since Jan 2020 and till the project end date (August 2020).

### Baseline characteristics of participants

Experimental and control groups were similar in sociodemographic and clinical characteristics at baseline, respectively, except more participants from the mindfulness group were females (Table 1). The mean (SD) age of participants was 64.5 (8.4) years, ranging from 40–88 years old, and 39 out of 68 were females (57.4%). Of 68 participants, mild PD (Hoehn and Yahr scale, stage 1–2) was seen in 27 participants (39.7%), and most (60.3%) had moderate PD (Hoehn and Yahr scale, stage 3). The mean (SD) MDS-UPDRS-III score was 34.8 (12.7) among all participants. At baseline, 21 out of 68 (30.9%) and 20 out of 68 (23.5%) presented with clinically significant anxiety symptoms and depressive symptoms, respectively, with a mean (SD) HADS score of 11.94 (6.5). Drop- (*n* = 7) and non-drop-out cases (*n* = 61) were similar in sociodemographic and clinical characteristics at baseline. Except that drop-out participants had a higher levodopa equivalent dose and better MoCA scores in naming and abstraction subscales (SupplementaryTable 1. Characteristics of drop-out and non-drop-out cases).

**Table 1:** Baseline socio-demographic characteristics of the participants (*n* = 68).

Characteristics	Participants, No. (%)			*P*-value
	All (*n* = 68)	MM (*n* = 33)	SRTE (*n* = 35)	
Age, mean (SD), years	64.5 (8.4)	62.7 (7.7)	66.1 (8.9)	0.094
Gender				
Male	29 (42.6)	10 (30.3)	19 (54.3)	0.046*
Female	39 (57.4)	23 (69.7)	16 (45.7)	
Marital status				
Single, separated, divorced, or widowed	17 (25%)	11 (33.3)	6 (17.1)	0.123
Married	51 (75%)	22 (66.7)	29 (82.9)	
BMI	22.8 (4.1)	35.2 (23.6)	30.9 (21.9)	0.089
Education				
Illiterate/Primary	14 (20.6)	7 (21.2)	7 (20)	0.902
Secondary or above	54 (79.4)	26 (78.9)	28 (80)	
Religion				
Christianity	10 (14.7)	4 (12.1)	6 (17.1)	0.767
Catholicity	6 (8.8)	4 (12.1)	2 (5.7)	
Buddhism	9 (13.2)	4 (12.1)	5 (14.3)	
No religion	43 (63.2)	21 (63.6)	22 (62.9)	
Living status				
Alone	10 (14.7)	5 (15.2)	5 (14.3)	0.920
With spouse, family, or friends	58 (85.3)	28 (84.8)	30 (85.7)	
Social security allowance	36 (52.9)	21 (63.6)	15 (42.9)	0.219
Disease staging				
Mild PD, H&Y stage I or II	27 (39.7)	17 (51.5)	10 (28.6)	0.053
Moderate PD, H&Y stage III	41 (60.3)	16 (48.5)	25 (71.4)	
Levodopa equivalent dose, mean (SD)	638.8 (593.4)	766.4 (607.2)	518.5 (562.3)	0.085
Charlson Comorbidity Index ≥1	8	4	4	1.0
Myocardial infarction	1 (1.5)	0 (0)	1 (2.9)	0.515
Congestive heart failure	2 (2.9)	1 (3.0)	1 (2.9)	0.739
Peripheral vascular disease	1 (1.5)	1 (3.0)	0 (0)	0.485
Mild liver disease	1 (1.5)	0 (0)	1 (2.9)	0.515
Diabetes without chronic complications	3 (4.4)	2 (6.1)	1 (2.9)	0.478
Tumor without metastases	2 (2.9)	1 (3.0)	1 (2.9)	0.739
Schwab and England Scale	8.8 (1.9)	8.8 (2.0)	8.9 (1.8)	0.965

Categorical and continuous variables were compared between the two groups using chi-square test, fisher’s exact test, and Independent *t*-test, respectively. Data are presented as mean (standard deviation), or *n* (%).

Comorbid conditions were assessed using Charlson Comorbidity Index.

*H&Y stage* Hoehn and Yahr stage, *MM* mindfulness meditation, *SRTE* stretching and resistance training exercise.

### Co-primary outcomes

For depressive symptoms, there was a significant difference between groups at T1 end point (*p* = 0.019) (Table 2). Compared with the SRTE group, the mindfulness group demonstrated significantly greater improvement in depressive symptoms at T1 with a small-to-moderate effect size of 0.37 (time-by-group interaction, T1: *β*, −1.13 [95% CI, −2.06, −0.19; *p* = 0.019]), whereas no significant difference between groups at T2 (T2: *β*, 0.83 [95% CI, −0.42, 2.06; *p* = 0.191]). In the mindfulness group, significant improvement within the group in depressive symptoms (T1: *d* = −0.48, *p* = 0.002) was found with a mean (SD) difference of −1.36 (0.37), whereas no significant difference within the SRTE group at either end points (T1: *d* = 0.07, *p* = 0.388; T2: *d* = 0.03, *p* = 0.71).

**Table 2:** Analysis of GEE for the comparison of outcomes (*n* = 68).

Outcomes	Mean (SD)		Effect Size (within group change from baseline)				Group × Time Interaction Effect^a^		
	MM	STRE	MM	*p*	STRE	*p*	β (95% CI)	*Effect size*	*p*
	HADS-anxiety								
T0	6.24 (3.46)	5.51 (3.80)							
T1	6.12 (3.14)	5.54 (3.81)	−0.04	0.734	0.01	0.941	−0.15 (−1.19, 0.88)	0.17	0.77
T2	6.66 (3.80)	6.28 (3.82)	0.12	0.602	0.20	0.098	−0.35 (−1.56, 0.87)	0.10	0.58
	HADS-depression								
T0	5.97 (2.95)	6.17 (3.57)							
T1	4.59 (2.75)	5.91 (4.18)	−0.48	0.002**	0.07	0.388	−1.13 (−2.06, −0.19)	0.37	0.019*
T2	6.91 (3.26)	6.29 (3.45)	0.30	0.110	0.03	0.710	0.83 (−0.42, 2.06)	0.18	0.191
	FFMQ-observing								
T0	12.97 (2.84)	12.57 (3.37)							
T1	13.52 (3.37)	12.54 (3.47)	0.18	0.122	−0.01	0.958	0.08 (−1.63, 1.78)	0.29	0.381
T2	12.52 (3.31)	12.04 (3.71)	−0.15	0.601	−0.15	0.446	0.58 (−0.72, 1.89)	0.14	0.929
	FFMQ-describing								
T0	13.39 (3.26)	13.11 (3.47)							
T1	13.72 (3.56)	12.77 (3.29)	0.09	0.332	−0.10	0.472	0.67 (−0.52, 1.85)	0.28	0.271
T2	13.45 (3.64)	12.24 (3.35)	−0.02	0.795	−0.26	0.092	0.94 (−0.50, 2.38)	0.35	0.202
	FFMQ-acting with awareness								
T0	12.45 (3.85)	14.03 (2.93)							
T1	13.37 (3.65)	13.97 (3.03)	0.25	0.163	−0.02	0.899	0.98 (−0.48, 2.43)	0.18	0.190
T2	12.76 (3.44)	13.97 (3.21)	0.08	0.377	−0.02	0.781	0.37 (−0.84, 1.57)	0.36	0.551
	FFMQ-non-judging								
T0	11.70 (2.53)	11.71 (3.12)							
T1	12.37 (2.55)	12.60 (2.74)	0.26	0.213	0.30	0.121	−0.22 (−1.63, 1.20)	0.90	0.765
T2	12.91 (2.58)	12.38 (2.63)	0.48	0.022*	0.23	0.177	0.54 (−0.75, 0.69)	0.20	0.407
	FFMQ-non-reacting								
T0	12.97 (2.17)	12.80 (2.97)							
T1	13.21 (2.85)	12.23 (2.99)	0.09	0.598	−0.19	0.084	0.81 (−0.31, 1.93)	0.34	0.156
T2	13.26 (3.28)	11.18 (2.96)	0.10	0.793	−0.55	0.001***	1.91 (0.54, 3.27)	0.67	0.006**
	FFMQ-total								
T0	63.48 (1.53)	64.23 (1.30)							
T1	66.00 (1.76)	64.11 (1.33)	1.53	0.010**	0.09	0.074	2.62 (−0.79, 6.05)	2.44	0.132
T2	66.33 (1.69)	61.79 (1.69)	1.78	0.026	1.62	0.582	3.28 (−0.42, 6.98)	2.67	0.082
	MDS-UPDRS-III								
T0	34.76 (11.83)	34.74 (13.29)							
T1	32.51 (11.44)	32.36 (11.91)	−0.19	0.095	−0.19	0.010**	0.132 (-2.86, 3.12)	0.01	0.931
	TUG								
T0	14.29 (11.16)	12.94 (3.79)							
T1	12.61 (6.63)	12.28 (3.83)	−0.18	0.207	−0.17	0.123	−1.02 (−3.12, 1.07)	0.06	0.338
	HK-MoCA-visuospatial								
T0	3.82 (1.19)	4.14 (1.02)							
T1	4.11 (1.01)	4.30 (1.21)	0.27	0.199	0.14	0.419	0.14 (−0.35, 0.63)	0.17	0.575
	HK-MoCA-naming								
T0	2.85 (0.36)	2.89 (0.40)							
T1	2.85 (0.37)	2.85 (0.50)	0.01	1.000	0.07	0.572	0.03 (−0.11, 0.17)	0	0.680
	HK-MoCA-attention								
T0	5.27 (0.93)	5.37 (0.64)							
T1	5.45 (0.64)	5.52 (0.63)	0.22	0.442	0.23	0.282	0.03 (−0.38, 0.44)	0.11	0.887
	HK-MoCA-language								
T0	2.82 (0.39)	2.77 (0.48)							
T1	2.80 (0.41)	2.83 (0.46)	−0.04	0.662	0.12	0.325	−0.07 (−0.26, 0.12)	0.07	0.461
	HK-MoCA-abstraction								
T0	1.45 (0.56)	1.43 (0.60)							
T1	1.43 (0.58)	1.34 (0.60)	0.05	1.000	−0.16	0.374	0.067 (−0.21, 0.35)	0.15	0.640
	HK-MoCA-delayed recall								
T0	3.58 (1.46)	3.06 (1.84)							
T1	4.10 (1.30)	3.77 (1.25)	0.36	0.045	0.45	0.008**	−0.19 (−0.81, 0.43)	0.26	0.547
	HK-MoCA-orientation								
T0	5.73 (0.62)	5.63 (0.59)							
T1	5.96 (0.19)	5.88 (0.42)	0.52	0.103	0.49	0.037	−0.01 (−0.34, 0.31)	0.25	0.936
	HK-MoCA-total								
T0	25.73 (3.06)	25.49 (3.32)							
T1	26.77 (2.61)	26.70 (3.33)	0.37	0.006**	0.36	0.011*	−0.17 (−1.22, 0.89)	0.02	0.757
	PDQ-8 summary index								
T0	33.43 (15.76)	26.70 (18.44)							
T1	31.73 (15.19)	29.11 (17.58)	−0.11	0.284	0.13	0.136	−4.11 (−8.28, 0.05)	0.16	0.053
T2	36.26 (17.58)	28.17 (15.45)	0.17	0.287	0.09	0.547	1.35 (−4.86, 7.57)	0.49	0.669

The control group and the baseline measurement were chosen as the reference categories in the GEE model and its corresponding dummy variables were group = 1 and time = 0.

Mean (SD) = change of mean (SD) from T1 and T2 in relation to T0, respectively.

*Exp* experimental group, *Control* control group, *MDS-UPDRS-III* Movement Disorders Society United Parkinson’s Disease Rating Scale Part III, *MM* mindfulness meditation, *HADS* Hospital Anxiety and Depression Scale, *PDQ-8* Parkinson’s Disease Questionnaire-8, *SRTE* stretching and resistance training exercise, *T0* baseline, *T1* 8 weeks (immediate post-intervention), *T2* 20 weeks (three months post-intervention), CI confidence interval.

*p value ≤ 0.025; **p value ≤ 0.01; ***p value ≤ 0.001.

^a^Group*time effect: T1 and T2 = additional change of scores of intervention group compared with control group at T1& T2, respectively.

For anxiety, no significant difference between groups at either time points (T1: *β*, −0.15 [95% CI, −1.19, 0.88; *p* = 0.77]; T2: *β*, −0.35 [95% CI, −1.56, 0.87, *p* = 0.58]). No significant differences within both groups in the anxiety scores across time points.

### Secondary outcomes

The mindfulness group showed significant improvement compared with SRTE group in non-reacting aspect of mindfulness at T2 (*β*, 1.91 [95% CI, 0.54, 3.27; *p* = 0.006]) and marginally significant improvement in QoL at T1 (*β*, −4.11 [95% CI, −8.28, 0.05; *p* = 0.053]). For the mindfulness group, significant improvement was noted in the overall mindfulness (T1: *d* = 1.53, *p* = 0.010) and overall cognitive performance at T1 (T1: *d* = 0.37, *p* = 0.006), as well as increased non-judging aspect of mindfulness at T2 (T2: *d* = 0.48, *p* = 0.022). For the SRTE group, significant improvement was noted in MDS-UPDRS-III motor scores (T1: *d* = −0.19, *p* = 0.01), delayed recall (T1: *d* = 0.46, *p* = 0.008), and overall cognitive function (T1: *d* = 0.36, *p* = 0.011) at T1; whereas significant deterioration was noted in non-reacting aspect of mindfulness at T2 (*d* = −0.55, *p* = 0.001).

### Adverse events

A participant (3%) from the mindfulness group reported temporary back pain after the 7^th^ session. It was resolved by using of prop (placing a support cushion on the chair) and occasionally doing intermittent changes in body postures during the meditation practice; no medical attention was needed. Another participant (2.9%) from the SRTE group reported temporary mild knee pain during and after squatting exercises, however, no medical attention was required. No further serious adverse events were noted. No adverse events/accidents have been reported when participants accessed to community centers from their home.

### Feedback from participants

Supplementary Table 2 summarizes the findings of the satisfaction evaluation. All participants were satisfied with the study. Regarding the open-ended question, surprisingly, 67.7% and 51.6% of participants from the mindfulness group reported improvement in their constipation and sleep outcomes, which were not quantitatively assessed in this study. Regardless of the satisfactory attendance (overall attendance = 82.5%) and acceptable attrition (overall attrition = 7.3% at T1 face-to-face follow-up, 10.3% at T2 telephone follow-up), 60% of the participants reported safety concerns on their way to join the center-based activities caused by the uncertainty and threats of social unrests.

## Discussion

We observed that the mindfulness meditation group had greater improvement in psychospiritual outcomes than the conventional physical exercise group, which includes improved depressive symptoms at T1 and maintained non-reactivity at T2. In addition, the mindfulness group showed improved mindfulness and overall cognitive performance at T1 and increased non-judging awareness at T2, whereas the physical exercise group showed improved motor symptoms, delayed recall and overall cognitive performance at T1, with no statistically significant superiority noted. The effects of mindfulness meditation in improving immediate depressive symptoms were statistically significant with a small-to-moderate in size, and reaching marginal clinical significance in PD patients. Significant emotional distress or mental disorder among PD populations during and after large-scale social movements and COVID-19 pandemic was evident^14,15^, which is associated with higher subsequent psychiatric symptoms^16^. It shall be noting that the mindfulness program was not specifically designed to address the stress triggers owing to social unrest and/or COVID-19 pandemic, the treatment effects and between-group differences on psychospiritual outcomes would likely to be reduced. Given the unfavorable and unexpected stress triggers, the observed improvements were confirmative because interventions and assessments were conducted during the strikes of social unrest and/or the COVID-19 pandemic.

It is worth noting that the emotional reactivity of exercise group has significantly worsen at T2 (during the start of COVID pandemic) and such difference remained statistically significant when compared to the mindfulness meditation group. Previous neuroimaging studies have suggested that individuals who showed less openness and acceptance to experiences, exhibited stronger activation in limbic systems when labeling negative thoughts, stressful events and experiences^17^. Hence, anti-pandemic measures should consider providing mindfulness tips/techniques to ease the public emotional reactivity and mitigate pandemic-related distress, which in turn might help to inhibit habitual responses contributing to sharing of false information and ineffective coping.

Furthermore, emerging evidence indicated that the prolonged public health and virus-control measures implemented to combat the COVID-19 pandemic severely disrupted various aspects of daily life among the PD population worldwide, particularly in overcrowded cities like Hong Kong^18^. Virus-control measures implemented were as follows: social lockdown, closures of community-based leisure and recreational facilities, and suspension of rehabilitation, and home-based care services. The threat to health and the economic downturn associated with the COVID-19 pandemic incur a tremendous burden on the population’s mental health^19^. All these suggested that PD patients were at high risk of psychological distress during this period. Considering that stress was identified as a risk factor for PD progression and cognitive impairment, our study suggests integrating mindfulness training into evidence-based rehabilitation protocols. Psychologically informed physical therapy could be considered a favorable complementary behavioral approach to strengthening physical functions, psychospiritual resilience and cognitive reserve of PD patients, thereby, reducing their risk of psychiatric or cognitive comorbidities. As for future research directions, further efforts should be expended to test and translate center-based behavioral rehabilitation practices into home-based settings. A hybrid delivery mode is preferred to address the challenges imposed by the common barriers in participating in center-based rehabilitation and any future social unrests/infectious disease outbreaks.

Compared to our previous clinical trial of mindfulness yoga, there was inconsistency in the evidence of its effects on anxiety, motor symptoms, and HRQOL^12,20,21^. Such discrepancies might be due to the different teaching/training approaches of mindfulness practices, as well as confounding factors related to the external stressors. In the current study, the program aimed to cultivate mindfulness via meditation and breathing techniques. The training was relatively static and started with focused attention on an object to develop attentional stability and non-judgmental awareness of the current mental state^22^. This mentally-oriented approach might be particularly challenging for some participants as dopamine depletion in PD might affect frontal functions, leading to executive dysfunction, impaired attention and increased feelings of distractions^23^. In comparison, the previous study cultivated mindfulness mainly via yoga, which is a relatively dynamic and physically exerting form of mind-body training^24^. On top of emotional and cognitive regulating pathway, we suspect the physically exerting yoga training might provide additional symptomatic relief (i.e. to improve stiffness, muscle weakness, and postural instability) and address additional stress triggers owing to physical symptoms. These findings suggest mindfulness training via physically exerting mind-body approach might multiply the avenue of physical and psychospiritual benefits in PD patients. However, the mechanistic effects of these different use of individual mindfulness practices are still unknown. Future studies should extend the investigation into the use of individual mindfulness techniques (i.e. cultivating mindfulness via meditation vs yoga), and to further examine their therapeutic value and underlying mediating mechanisms among PD patients, preferably using both subjective (i.e. validated patient-reported outcomes such as HADS) and objective outcome measures related to stress and inflammatory responses (i.e. cortisol, IL-6 and TNF-α)^25^. Such evidence is important to guide the development of personalized mindfulness therapy to optimize its therapeutic benefits.

Both groups showed significant improvement in delayed recall and overall cognitive performance among PD patients. These findings are consistent with a 2018 systematic review of exercise interventions^26^, which concluded moderate-to-strong evidence on moderate-to-vigorous intensity physical exercise. It improved not only motor symptoms, but also cognitive function in PD patients, including global cognitive function, processing speed, sustained attention, and mental flexibility. Pickut and colleagues^27^ investigated the effects of an 8-week mindfulness-based intervention (MBI) on anatomical brain MRI scans using a voxel-based morphometry approach, and found that PD patients who received MBI (*n* = 14) showed increased gray matter density in the amygdala, the caudate nucleus, and the left occipital lobe compared to usual care control (*n* = 13)^27^. In addition, MBI studies consistently showed increased activity and structural changes in the insula, anterior and posterior cingulate cortex, striatum, and the medial and dorsolateral prefrontal cortex^28^. All brain regions have important roles to play in executive functioning, memory, information processing, attentional control, emotional regulation, and self-awareness. Emerging empirical evidence showed that mindfulness may improve emotional and cognitive functioning in PD patients^29^. Indeed, cognitive impairment is a common non-motor complication of PD, and 50% of the patients demonstrated a significant cognitive decline in the first 3–5 years of illness^26^. A prospective 3-year longitudinal study with a total of 182 PD patients shows that mild cognitive impairment (MCI) at PD diagnosis predicts a highly increased risk for early dementia^30^. Therefore, early screening for MCI and evidence-based behavioral interventions for cognitive rehabilitation for at-risk PD patients, such as physical exercise and mindfulness training, should be prescribed to improve their cognitive functioning and prevent subsequent development of dementia^31^.

Based on our knowledge, this is the first clinical trial of MBI for PD conducted during social unrest and the COVID-19 pandemic. This trial provides invaluable insights as a guide for further development and implements mindfulness as a complementary therapeutic option for PD patients in coping with unplanned external stressors and major adversities. Study strengths include an assessor-masked, randomized clinical design with multiple follow-up time points to elucidate the residual effects of the interventions, involvement of an active control group, comprehensive measurement of physio-psycho-cognitive, and spiritual outcomes. The data analysis followed an intention-to-treat principle.

This study has limitations and must be acknowledged. Although the number of included patients was significantly smaller than originally planned because of the COVID-19 pandemic and associated nationwide regulations, several significant changes were still detected. Nevertheless, the sample size of the current study is above the average of existing MBI clinical trials for PD patients (number of studies = 7; range = 13–138; mean sample size = 50)^12,32–37^. We believe that these findings will provide valuable evidence to guide the future MBI research for PD. In addition, local government and university guidelines prohibited face-to-face non-clinical services and research activities at T2. Thus, we were unable to perform a face-to-face examination at T2, the sustained intervention effects on motor symptoms, functional mobility, and cognition remained unknown. Thirdly, we did not measure participants’ expectation regarding the effects of each intervention nor measuring their physical activity levels during the study period. Future clinical trial should assess participants’ expectations and physical activity levels as co-variables, which allows a better interpretation of the therapeutic outcomes between groups^38^. The incorporation of singing bowls might add a potential layer of complexity of the meditation effects. For instance, participants’ feedback collected from the satisfaction evaluation revealed improved constipation outcomes owing to the resonant vibrations generated by the singing bowls being felt by the body and intestines. Future study could consider adopting a qualitative approach to explore participants’ experience of meditation with and without sound catalyst. Furthermore, we observed a higher drop-out and lower attendance for the mindfulness meditation group compared to the physical exercise group. One possibility is that PD patients might be more interested to attend rehabilitation activities with physical training components. However, owing to the on-going protests that occurred during the intervention period, safety concerns while traveling to community center might have an impact on the intervention compliance. Lastly, selection bias may arise because the study participants were enrolled through convenience sampling. There were more females (57%) than males that participated in this study, while PD is 1.5 times more common in males than in females. Such pattern may indicate that women with PD might be more active in reaching out to community resources and more willing to join group-based rehabilitation services. We excluded people who had severe motor limitations, uncontrolled/active psychiatric disorders, or significant cognitive impairment. Thus, the PD population is only partially represented. All these factors may limit the generalizability of the study findings to the entire PD population.

As compared with physical exercises, mindfulness meditation appeared to be a feasible strategy for managing depressive symptoms and maintaining emotional stability, with comparable benefits in cognitive performance, among patients with mild-to-moderate PD. These findings suggest that mindfulness meditation might be a promising complementary lifestyle practice for cultivating non-reactivity and managing depressive mood among patients with PD, which exerts similar benefits on cognition as compared to conventional physical exercise. Given the additional stress triggers related to the unfavorable external conditions (i.e. social unrest and COVID-19 pandemic) which were likely to reduce the treatment effects, such improvements on psychospiritual outcomes are confirmative. Considering that PD is not just a psychologically distressing life event but is also associated with physical and cognitive impairment, health care professionals should integrate mindfulness into evidence-based exercise prescription for PD rehabilitation. Further investigation is warranted to establish the mechanistic effect and compliance of various forms of mindfulness practices. Alternative delivery modes including telehealth should also be included in the further investigation given social unrest and/or infectious disease outbreaks^39–41^.

## Methods

### Study design

This assessor-masked, multicentered, randomized clinical trial (RCT) was conducted among PD patients between August 1, 2019 and February 28, 2020 comparing mindfulness meditation with an active exercise control (stretching and resistance training exercise). The trial has been prospectively registered in the HKU Clinical Trials Registry identifier: HKUCTR-2681. Recruitment was conducted via convenience sampling at two out-patient neurology clinics of regional public hospitals and via the Community Rehabilitation Network. Meanwhile, the study implementation was conducted at three community wellness centers. Ethical approval was obtained from the Institutional Review Board of the University of Hong Kong/Hospital Authority Hong Kong West Cluster (HKU/HA HKW IRB) (Reference Number: UW 19-446). All participants provided written informed consent and all obtained data were anonymous. This report followed the Consolidated Standards of Reporting Trials (CONSORT 2010) guidelines and its extension to non-pharmacological interventions^42^. Ethics approval for this study was obtained from the Institutional Review Board of the University of Hong Kong/ Hospital Authority Hong Kong West Cluster (HKU/HA HKW IRB) (Reference Number: UW 19-446).

### Study participants

Individuals were eligible to participate and included in the trial if they were: (i) diagnosed with idiopathic PD (Hoehn and Yahr stage II-IV); (ii) aged ≥18 years; and (iii) able to give written consent. Individuals were excluded if they were: (i) regularly practicing meditation once a week or more during the past 6 months; (ii) currently receiving pharmacological treatments (such as antidepressants) or surgical treatments (such as deep brain stimulation) for uncontrolled/active psychiatric disorders (such as including schizophrenia, psychosis, major depressive disorder, etc.); (iii) were currently participating in any other behavioral or pharmacological trial; (iv) screened with significant cognitive impairment as indicated by the abbreviated mental test lower than 6; or (v) had serious medical conditions except for PD or other contraindication that might hinder their full participation to the intervention (e.g., severe hearing or vision impairment, etc.).

### Sampling and recruitment strategies

Subjects were recruited via referrals from two major sources: (i) neurological outpatient clinics from two local regional hospitals, which are the Queen Mary Hospital and Pamela Youde Nethersole Eastern Hospital; and (ii) three PD support groups, which the Community Rehabilitation Network (CRN), Hong Kong Parkinson’s Disease Association (HKPDA), and Hong Kong Parkinson’s Disease Foundation (HKPDF). The intervention was conducted in three community wellness centers to enhance the sample representativeness and participants’ accessibility, recruitment, and adherence. Community wellness is located in three major districts in Hong Kong, namely, Hong Kong-West, Hong Kong-East, and Kowloon. Combinations of recruitment strategies were used, including electronic mailing, printed flyers within the patient support centers, and neurological clinics, and newsletters disseminated among patient support groups. All promotional materials and registration were made accessible online.

### Allocation concealment, assignment of interventions and blinding

Prescreening was done via telephone calls and in neurology clinics, and participants who met the criteria underwent baseline assessments. Afterward, participants were randomly assigned to experimental or control groups at a ratio of 1:1 through a computerized permuted block randomization generator, in which randomized sizes of 4, 6, and 8 were used. The randomization sequence was generated by an independent research coordinator and the group assignments were concealed on cards placed inside sequentially numbered, sealed opaque envelopes until the assignment occurred. Outcome assessors were blinded to subject allocation and all assessments were conducted at CRN centers.

### Interventions

#### Mindfulness meditation

The mindfulness meditation program was adapted from the structured eight-week mindfulness-based stress reduction (MBSR) program conceptualized by Jon Kabat-Zin^43^ and our previous experiences of teaching mindfulness among local PD patients^12,20^, and was delivered in neutral terminology free from any particular religious affiliation. Previous PD literature reported that frequent and lengthy mind-body interventions (more than 3 times per week, with a total time >180 min per week) were associated with reduced effectiveness owing to the decreased adherence and increased physical intolerance experienced by PD patients, and suggested a moderate dose of about 120 min per week as a feasible and practical mind-body practice for PD patients^10^. Hence, compared to the standard MBSR program (eight weekly 2.5-hour sessions, one all-day session between sessions 6 and 7, and daily 45-min homework), the tested mindfulness meditation program was modified to eight 1.5-hour sessions, and 20-min home-based practice twice a week. The weekly session consisted of standardized core elements of mindfulness practices: (1) body scan, (2) guided meditation focusing one’s attention on the breath, (3) guided meditation facilitated by singing bowls with focus on being aware of bodily sensations and non-judgmental awareness, and (4) practicing being fully aware during everyday activities by using the breath as an anchor for the attention (Supplementary Table 3. Overview of the modified mindfulness meditation program). Each session started with 15-min of introduction and sharing, followed by 60-min of guided mindfulness meditation (30-min was facilitated with the use of singing bowls) and mindful breathing techniques, and 15-min of closing and sharing. Owing to the physical symptoms of PD patients including tremor, rigidity and bradykinesia, prolonged holding of the same pose is not recommended^44^. Hence, verbal cues of gentle movements were incorporated every 10-min during the guided meditation practice, so as to avoid exacerbation of physical symptoms. To compensate for the impaired attention and cognition in PD patients, 30-min of the guided meditation practice was facilitated with the use of singing bowls. Previous research has highlighted the combinations of high and low frequency sounds generated by singing bowls could produce resonant vibrations as a catalyst and sensational stimuli for mental activation to induce a deep relaxation response and immersive experiences of meditation^45,46^. During the guided meditation facilitated by singing bowls, participants were instructed to lie down on yoga mats, in a half-circle around the room with their heads pointed towards the singing bowls, which were placed on the floor near their heads. Participants were instructed to focus their awareness on sound and vibrations created and sensations felt by the body, and merely observe non-judgmentally for any sensations, emotions, or thoughts that may arise during the meditation practice.

#### Conventional stretching and resistance training exercise

This group of participants received weekly 90-min sessions of full-body physical exercise for 8 weeks. In addition, all participants were encouraged to perform 20-min home-based practice twice a week. The physical exercise protocol consisted of a progressive set of warm-ups, stretching, and resistance training exercises (SRTE) with moderate intensity, and cool-down exercises. The protocol was reviewed by two physiotherapists to confirm the validity for patients with PD, its safety has been demonstrated in our previous published trial^12^.

The purpose of adopting an active exercise control group was two-fold: (i) to counteract the confounding effects of regular social interaction among peers and instructors during weekly gatherings; and (ii) physical exercise is a standard treatment to provide PD patients additional symptomatic relief on top of pharmacotherapy. The format of interventions was comparable (group), contact time (8 weekly 90-minute sessions), number of participants per group (15–20 participants per session), and venue (activity rooms in community wellness centers). Each intervention was delivered to the participants according to a manualized protocol in which all instructors were trained. Mindfulness meditation was delivered by an instructor with MBSR teacher qualifications, whereas physical exercise was delivered by a qualified fitness instructor. All instructors were experienced in teaching people with chronic illnesses. All participants in each group of interventions were given information booklets covering instructions for home practice.

### Data collection and outcome measures

Outcome assessors were trained and masked to group allocation. Each participant was invited to the nearby community rehabilitation center to conduct a face-to-face clinical assessment and interview. If indicated, all assessments were conducted during the “on” state of medications to minimize motor fluctuations among participants. There were three assessment time points, including baseline (T0), immediate post-intervention (T1, 8 weeks), and three-month post-intervention (T2, 20 weeks). Telephone follow-up was offered as an alternative option for those who declined face-to-face clinical assessment to collect self-reported outcomes.

The primary outcome, psychological distress in terms of anxiety, and depressive symptoms were measured by the validated Hospital Anxiety and Depression Scale (HADS) (Chinese-Cantonese)^47,48^. HADS is a self-report questionnaire consisting of anxiety and depression subscales. Each subscale consists of seven items and each item is rated on a four-point scale. A high score represents a high level of psychological distress. HADS has been suggested to be utilized in the PD population because somatic symptoms that may potentially overlap Parkinsonian manifestations are not assessed on this scale^49^. In addition, HADS focuses on measuring the negative emotions of anxiety and depression, which were reported as the most prominent psychological factors among PD patients. The Cronbach alphas for the HADS-anxiety and HADS-depression subscales were 0.86 and 0.78. The test-retest reliability for subscales, as assessed by intraclass correlation coefficient (ICC), were 0.86 and 0.84 for the HADS-anxiety and HADS-depression^50^. Although no published data regarding minimal clinically important difference of HADS score was reported in PD, previous literature reported the MCID for the HADS, triangulated from the distribution-based, anchor-based, and Delphi-based findings, was 1.7 points in cardiovascular diseases^51^, meanwhile, the MCID of subscale scores for anxiety and depression were 1.32 and 1.40 in pulmonary diseases, respectively^52^.

Secondary outcomes included were: (i) PD-related motor symptoms as measured using the validated Movement Disorder Society-Unified Parkinson’s Disease Rating Scale, part III motor examination (MDS-UPDRS-III) (Chinese version)^53^, which covers domains related to tremor, rigidity, bradykinesia, gait, and postural instability; (ii) mobility was measured based on the Timed-up-and-go (TUG) test by the United States Food and Drug Administration approved EncephaLog application using a smartphone device^54^; (iii) cognition as measured by the Montreal Cognitive Assessment (MoCA) protocol (Hong Kong version)^55^, which consists of seven subtests that examine varying cognitive domains, including visuospatial/executive, naming, memory, attention, language, abstraction, and orientation; (iv) mindfulness as measured using the 20-item Five-Facet Mindfulness Questionnaire (Short-form) (FFMQ-SF) (Chinese version), which assesses five mindfulness domains including observing, describing, acting with awareness, nonjudgment of inner experience, and non-reaction to inner experience. FFMQ-SF has been validated in community adults recruiting from out-patient clinics (*n* = 156) and community settings (*n* = 230) which shared similar socio-demographic characteristic as compared to our study participants (Cronbach’s alpha = 0.80–0.83)^56^; and (v) HRQOL as assessed using the validated Parkinson’s Disease Questionnaire-8 (PDQ-8) (Chinese version)^57,58^, which yields a summary index score that captures disease-specific HRQOL in terms mobility, activities of daily living, emotional wellbeing, social support, cognitions, communication, bodily discomfort and stigma.

### Satisfactory, safety and adverse event

A satisfactory evaluation questionnaire (10-item) was administered to both groups at the last intervention session to collect feedback and perceived effects of the study. Adverse events were identified during the intervention sessions and by follow-up interview questions about significant intervention-induced discomfort, pain, or harm. Participants were instructed to inform the research team if they encountered any adverse event related to the study.

### Sample size

A moderate effect size of 0.59 was reported for people who presented with depressive symptoms according to a meta-analysis of mindfulness interventions compared with exercises^59^. Assuming an attrition rate of 15%, a total sample size of 112 participants was required in order to have 80% power to determine an effect of at least 0.59 at a 5% level of significance. We have originally planned three batches of intervention sessions during the project period (August 2019–2020) (1^st^ batch: Sept-Oct 2019; 2^nd^ batch: Jan-Feb 2020; 3^rd^ batch: May-Jun 2020). However, given the local government’s enforcement of stringent public health measures toward COVID-19, which include mandatory closing of all community-based leisure and recreational facilities and suspending research and non-clinical services since Jan 2020, all recruitment and community group-based rehabilitation sessions were suspended since Jan 2020 and till the project end date (August 2020). Therefore, only a total of 68 participants (61% of the proposed sample size) were included in this study.

### Statistical analysis

The socio-demographic and clinical outcomes of the participants at each time point were summarized using descriptive statistics. We applied the intention-to-treat (ITT) principle under which all participants were included in the analysis as randomized. The changes of the primary and secondary outcomes at immediate post-intervention (T1, 8 weeks) and three-month post-intervention (T1, 20 weeks) from baseline (T0) between the mindfulness meditation and SRTE groups were compared by the generalized estimating equations (GEE). The extra-covariance among the repeated measurements at T1 and T2 were accounted by a first-order auto-regressive working covariance matrix. The covariates included baseline value, group, time and group by time interaction. The GEE possessed the virtue of including participants in the analysis provided they had at least one follow-up measurements. Nevertheless, drop- and non-drop-out cases were compared to check for any differences in demographic characteristics and health conditions among PD participants. IBM SPSS 25 software was used for statistical analysis. The primary objectives of this study are to examine the immediate post-intervention effects of mindfulness meditation on anxiety and depression compared to stretching and resistance training exercise. To account for multiplicity, we consider 2.5% as the nominal level of significance by Bonferroni adjustment.

### Reporting summary

Further information on research design is available in the Nature Research Reporting Summary linked to this article.

## Supplementary information

Reporting Summary

Supplementary table 1; Supplementary table 2; Supplementary table 3

## Data Availability

Individual participant data that underlie the results reported in this article, after deidentification (text, tables, and figures), are available for researchers who provide a methodologically sound proposal with ethics approval. Proposal should be directed to the corresponding author (email: jojo.yykwok@gmail.com). To gain access, data requestors will need to sign a data access agreement. Data are available for 5 years.
