# Supplementary material for: A randomized clinical trial of mindfulness meditation versus exercise in Parkinson’s disease during social unrest
Source: NPJ Parkinsons Dis. 2023 Jan 21;9:7. doi: 10.1038/s41531-023-00452-w (PMC9862216; doi:10.1038/s41531-023-00452-w)
Supplement: Supplementary file 1 — Reporting Summary [file 41531_2023_452_MOESM1_ESM.pdf]

nature portfolio

Corresponding author(s): Jojo Yan Yan KWOK

Last updated by author(s): Dec 23, 2022

Reporting Summary

Nature Portfolio wishes to improve the reproducibility of the work that we publish. This form provides structure for consistency and transparency in reporting. For further information on Nature Portfolio policies, see our Editorial Policies and the Editorial Policy Checklist.

Statistics

For all statistical analyses, confirm that the following items are present in the figure legend, table legend, main text, or Methods section.

n/a	Confirmed	
☐	☒	The exact sample size (n) for each experimental group/condition, given as a discrete number and unit of measurement
☐	☒	A statement on whether measurements were taken from distinct samples or whether the same sample was measured repeatedly
☐	☒	The statistical test(s) used AND whether they are one- or two-sided<br>*Only common tests should be described solely by name; describe more complex techniques in the Methods section.*
☐	☒	A description of all covariates tested
☐	☒	A description of any assumptions or corrections, such as tests of normality and adjustment for multiple comparisons
☐	☒	A full description of the statistical parameters including central tendency (e.g. means) or other basic estimates (e.g. regression coefficient) AND variation (e.g. standard deviation) or associated estimates of uncertainty (e.g. confidence intervals)
☐	☒	For null hypothesis testing, the test statistic (e.g. F, t, r) with confidence intervals, effect sizes, degrees of freedom and P value noted<br>*Give P values as exact values whenever suitable.*
☒	☐	For Bayesian analysis, information on the choice of priors and Markov chain Monte Carlo settings
☐	☒	For hierarchical and complex designs, identification of the appropriate level for tests and full reporting of outcomes
☐	☒	Estimates of effect sizes (e.g. Cohen's d, Pearson's r), indicating how they were calculated

Our web collection on statistics for biologists contains articles on many of the points above.

Software and code

Policy information about availability of computer code

Data collection	Google form, Excel.
Data analysis	IBM SPSS 25 software.

For manuscripts utilizing custom algorithms or software that are central to the research but not yet described in published literature, software must be made available to editors and reviewers. We strongly encourage code deposition in a community repository (e.g. GitHub). See the Nature Portfolio guidelines for submitting code & software for further information.

Data

Policy information about availability of data

All manuscripts must include a data availability statement. This statement should provide the following information, where applicable:

- Accession codes, unique identifiers, or web links for publicly available datasets
- A description of any restrictions on data availability
- For clinical datasets or third party data, please ensure that the statement adheres to our policy

Individual participant data that underlie the results reported in this article, after deidentification (text, tables, and figures), are available for researchers who provide a methodologically sound proposal with ethics approval. Proposal should be directed to the corresponding author (email: jojo.yykwok@gmail.com). To gain access, data requestors will need to sign a data access agreement. Data are available for 5 years.

Human research participants

Policy information about studies involving human research participants and Sex and Gender in Research.

Reporting on sex and gender	Sex of participants was determined based on self-report. No sex-based analyses have been performed.
Population characteristics	Sociodemographic and clinical characteristics of all participants were shown in Table 1.
Recruitment	Recruitment was conducted via convenience sampling at two out-patient neurology clinics of regional public hospitals and via the Community Rehabilitation Network in Hong Kong. Selection bias may arise because the study participants were enrolled through convenience sampling. There were more females (57%) than males that participated in this study, while PD is 1.5 times more common in males than in females. Such pattern may indicate that women with PD might be more active in reaching out to community resources and more willing to join group-based rehabilitation services. We excluded people who had severe motor limitations, uncontrolled/active psychiatric disorders, or significant cognitive impairment. Thus, the PD population is only partially represented. All these factors may limit the generalizability of the study findings to the entire PD population.
Ethics oversight	Ethics approval for this study was obtained from the Institutional Review Board of the University of Hong Kong/ Hospital Authority Hong Kong West Cluster (HKU/HA HKW IRB) (Reference Number: UW 19-446).

Note that full information on the approval of the study protocol must also be provided in the manuscript.

Field-specific reporting

Please select the one below that is the best fit for your research. If you are not sure, read the appropriate sections before making your selection.

☐ Life sciences ☒ Behavioural & social sciences ☐ Ecological, evolutionary & environmental sciences

For a reference copy of the document with all sections, see nature.com/documents/nr-reporting-summary-flat.pdf

Life sciences study design

All studies must disclose on these points even when the disclosure is negative.

Sample size	*Describe how sample size was determined, detailing any statistical methods used to predetermine sample size OR if no sample-size calculation was performed, describe how sample sizes were chosen and provide a rationale for why these sample sizes are sufficient.*
Data exclusions	*Describe any data exclusions. If no data were excluded from the analyses, state so OR if data were excluded, describe the exclusions and the rationale behind them, indicating whether exclusion criteria were pre-established.*
Replication	*Describe the measures taken to verify the reproducibility of the experimental findings. If all attempts at replication were successful, confirm this OR if there are any findings that were not replicated or cannot be reproduced, note this and describe why.*
Randomization	*Describe how samples/organisms/participants were allocated into experimental groups. If allocation was not random, describe how covariates were controlled OR if this is not relevant to your study, explain why.*
Blinding	*Describe whether the investigators were blinded to group allocation during data collection and/or analysis. If blinding was not possible, describe why OR explain why blinding was not relevant to your study.*

Behavioural & social sciences study design

All studies must disclose on these points even when the disclosure is negative.

Study description	Data are quantitative.
Research sample	Experimental and control groups were similar in sociodemographic and clinical characteristics at baseline, respectively, except more participants from the mindfulness group were females (Table 1). The mean (SD) age of participants was 64.5 (8.4) years, ranging from 40–88 years old, and 39 out of 68 were females (57.4%). Of 68 participants, mild PD (Hoehn and Yahr scale, stage 1–2) was seen in 27 participants (39.7%), and most (60.3%) had moderate PD (Hoehn and Yahr scale, stage 3).
Sampling strategy	Recruitment was conducted via convenience sampling at two out-patient neurology clinics of regional public hospitals and via the Community Rehabilitation Network in Hong Kong. Selection bias may arise because the study participants were enrolled through convenience sampling. There were more females (57%) than males that participated in this study, while PD is 1.5 times more common in males than in females. Such pattern may indicate that women with PD might be more active in reaching out to community resources and more willing to join group-based rehabilitation services. We excluded people who had severe motor

limitations, uncontrolled/active psychiatric disorders, or significant cognitive impairment. Thus, the PD population is only partially represented. All these factors may limit the generalizability of the study findings to the entire PD population.
A moderate effect size of 0.59 was reported for people who presented with depressive symptoms according to a meta-analysis of mindfulness interventions compared with exercises (59). Assuming an attrition rate of 15%, a total sample size of 112 participants was required in order to have 80% power to determine an effect of at least 0.59 at a 5% level of significance. We have originally planned three batches of intervention sessions during the project period (August 2019-2020) (1st batch: Sept-Oct 2019; 2nd batch: Jan-Feb 2020; 3rd batch: May-Jun 2020). However, given the local government's enforcement of stringent public health measures toward COVID-19, which include mandatory closing of all community-based leisure and recreational facilities and suspending research and non-clinical services since Jan 2020, all recruitment and community group-based rehabilitation sessions were suspended since Jan 2020 and till the project end date (August 2020). Therefore, only a total of 68 participants (61% of the proposed sample size) were included in this study.

Data collection

Outcome assessors were trained and masked to group allocation. Each participant was invited to the nearby community rehabilitation center to conduct a face-to-face clinical assessment and interview. If indicated, all assessments were conducted during the "on" state of medications to minimize motor fluctuations among participants. There were three assessment time points, including baseline (T0), immediate post-intervention (T1, 8 weeks), and three-month post-intervention (T2, 20 weeks). Telephone follow-up was offered as an alternative option for those who declined face-to-face clinical assessment to collect self-reported outcomes.

Timing

There were three assessment time points, including baseline (T0: 8-30 Aug, 2019), immediate post-intervention (T1, 8 weeks: 28 Oct-21 Nov, 2019), and three-month post-intervention (T2, 20 weeks: 7-18 Feb, 2020).

Data exclusions

No data was excluded.

Non-participation

Drop- (n = 7) and non-drop-out cases (n = 61) were similar in sociodemographic and clinical characteristics at baseline. Except that drop-out participants had a higher levodopa equivalent dose and better MoCA scores in naming and abstraction subscales (Supplementary table 1. Characteristics of drop-out and non-drop-out cases).

Randomization

Prescreening was done via telephone calls and in neurology clinics, and participants who met the criteria underwent baseline assessments. Afterward, participants were randomly assigned to experimental or control groups at a ratio of 1:1 through a computerized permuted block randomization generator, in which randomized sizes of 4, 6, and 8 were used.
33 and 35 participants were randomized to the experimental group and control group, respectively.

Ecological, evolutionary & environmental sciences study design

All studies must disclose on these points even when the disclosure is negative.

Study description

Briefly describe the study. For quantitative data include treatment factors and interactions, design structure (e.g. factorial, nested, hierarchical), nature and number of experimental units and replicates.

Research sample

Describe the research sample (e.g. a group of tagged Passer domesticus, all Stenocereus thurberi within Organ Pipe Cactus National Monument), and provide a rationale for the sample choice. When relevant, describe the organism taxa, source, sex, age range and any manipulations. State what population the sample is meant to represent when applicable. For studies involving existing datasets, describe the data and its source.

Sampling strategy

Note the sampling procedure. Describe the statistical methods that were used to predetermine sample size OR if no sample-size calculation was performed, describe how sample sizes were chosen and provide a rationale for why these sample sizes are sufficient.

Data collection

Describe the data collection procedure, including who recorded the data and how.

Timing and spatial scale

Indicate the start and stop dates of data collection, noting the frequency and periodicity of sampling and providing a rationale for these choices. If there is a gap between collection periods, state the dates for each sample cohort. Specify the spatial scale from which the data are taken

Data exclusions

If no data were excluded from the analyses, state so OR if data were excluded, describe the exclusions and the rationale behind them, indicating whether exclusion criteria were pre-established.

Reproducibility

Describe the measures taken to verify the reproducibility of experimental findings. For each experiment, note whether any attempts to repeat the experiment failed OR state that all attempts to repeat the experiment were successful.

Randomization

Describe how samples/organisms/participants were allocated into groups. If allocation was not random, describe how covariates were controlled. If this is not relevant to your study, explain why.

Blinding

Describe the extent of blinding used during data acquisition and analysis. If blinding was not possible, describe why OR explain why blinding was not relevant to your study.

Did the study involve field work? ☐ Yes ☐ No

Field work, collection and transport

Field conditions	*Describe the study conditions for field work, providing relevant parameters (e.g. temperature, rainfall).*
Location	*State the location of the sampling or experiment, providing relevant parameters (e.g. latitude and longitude, elevation, water depth).*
Access & import/export	*Describe the efforts you have made to access habitats and to collect and import/export your samples in a responsible manner and in compliance with local, national and international laws, noting any permits that were obtained (give the name of the issuing authority, the date of issue, and any identifying information).*
Disturbance	*Describe any disturbance caused by the study and how it was minimized.*

Reporting for specific materials, systems and methods

We require information from authors about some types of materials, experimental systems and methods used in many studies. Here, indicate whether each material, system or method listed is relevant to your study. If you are not sure if a list item applies to your research, read the appropriate section before selecting a response.

Materials & experimental systems

n/a	Involved in the study	
☒	☐	Antibodies
☒	☐	Eukaryotic cell lines
☒	☐	Palaeontology and archaeology
☒	☐	Animals and other organisms
☐	☒	Clinical data
☒	☐	Dual use research of concern

Methods

n/a	Involved in the study	
☒	☐	ChIP-seq
☒	☐	Flow cytometry
☒	☐	MRI-based neuroimaging

Antibodies

Antibodies used	*Describe all antibodies used in the study; as applicable, provide supplier name, catalog number, clone name, and lot number.*
Validation	*Describe the validation of each primary antibody for the species and application, noting any validation statements on the manufacturer's website, relevant citations, antibody profiles in online databases, or data provided in the manuscript.*

Eukaryotic cell lines

Policy information about cell lines and Sex and Gender in Research

Cell line source(s)	*State the source of each cell line used and the sex of all primary cell lines and cells derived from human participants or vertebrate models.*
Authentication	*Describe the authentication procedures for each cell line used OR declare that none of the cell lines used were authenticated.*
Mycoplasma contamination	*Confirm that all cell lines tested negative for mycoplasma contamination OR describe the results of the testing for mycoplasma contamination OR declare that the cell lines were not tested for mycoplasma contamination.*
Commonly misidentified lines (See ICLAC register)	*Name any commonly misidentified cell lines used in the study and provide a rationale for their use.*

Palaeontology and Archaeology

Specimen provenance	*Provide provenance information for specimens and describe permits that were obtained for the work (including the name of the issuing authority, the date of issue, and any identifying information). Permits should encompass collection and, where applicable, export.*
Specimen deposition	*Indicate where the specimens have been deposited to permit free access by other researchers.*

Dating methods | *If new dates are provided, describe how they were obtained (e.g. collection, storage, sample pretreatment and measurement), where they were obtained (i.e. lab name), the calibration program and the protocol for quality assurance OR state that no new dates are provided.*

☐ Tick this box to confirm that the raw and calibrated dates are available in the paper or in Supplementary Information.

Ethics oversight | *Identify the organization(s) that approved or provided guidance on the study protocol, OR state that no ethical approval or guidance was required and explain why not.*

Note that full information on the approval of the study protocol must also be provided in the manuscript.

Animals and other research organisms

Policy information about studies involving animals; ARRIVE guidelines recommended for reporting animal research, and Sex and Gender in Research

Laboratory animals | *For laboratory animals, report species, strain and age OR state that the study did not involve laboratory animals.*

Wild animals | *Provide details on animals observed in or captured in the field; report species and age where possible. Describe how animals were caught and transported and what happened to captive animals after the study (if killed, explain why and describe method; if released, say where and when) OR state that the study did not involve wild animals.*

Reporting on sex | *Indicate if findings apply to only one sex; describe whether sex was considered in study design, methods used for assigning sex. Provide data disaggregated for sex where this information has been collected in the source data as appropriate; provide overall numbers in this Reporting Summary. Please state if this information has not been collected. Report sex-based analyses where performed, justify reasons for lack of sex-based analysis.*

Field-collected samples | *For laboratory work with field-collected samples, describe all relevant parameters such as housing, maintenance, temperature, photoperiod and end-of-experiment protocol OR state that the study did not involve samples collected from the field.*

Ethics oversight | *Identify the organization(s) that approved or provided guidance on the study protocol, OR state that no ethical approval or guidance was required and explain why not.*

Note that full information on the approval of the study protocol must also be provided in the manuscript.

Clinical data

Policy information about clinical studies

All manuscripts should comply with the ICMJE guidelines for publication of clinical research and a completed CONSORT checklist must be included with all submissions.

Clinical trial registration | HKU Clinical Trials Registry identifier: HKUCTR-2681

Study protocol | http://www.hkuctr.com/Study/Show/734d7356535845ff8786c7c336ffed0a

Data collection | Outcome assessors were trained and masked to group allocation. Each participant was invited to the nearby community rehabilitation center to conduct a face-to-face clinical assessment and interview. If indicated, all assessments were conducted during the "on" state of medications to minimize motor fluctuations among participants. There were three assessment time points, including baseline (T0), immediate post-intervention (T1, 8 weeks), and three-month post-intervention (T2, 20 weeks). Telephone follow-up was offered as an alternative option for those who declined face-to-face clinical assessment to collect self-reported outcomes.

Outcomes | The primary outcome, psychological distress in terms of anxiety, and depressive symptoms were measured by the validated Hospital Anxiety and Depression Scale (HADS) (Chinese-Cantonese) (47,48). HADS is a self-report questionnaire consisting of anxiety and depression subscales. Each subscale consists of seven items and each item is rated on a four-point scale. A high score represents a high level of psychological distress. HADS has been suggested to be utilized in the PD population because somatic symptoms that may potentially overlap Parkinsonian manifestations are not assessed on this scale (49). In addition, HADS focuses on measuring the negative emotions of anxiety and depression, which were reported as the most prominent psychological factors among PD patients. The Cronbach alphas for the HADS-anxiety and HADS-depression subscales were 0.86 and 0.78. The test-retest reliability for subscales, as assessed by intraclass correlation coefficient (ICC), were 0.86 and 0.84 for the HADS-anxiety and HADS-depression (50). Although no published data regarding minimal clinically important difference of HADS score was reported in PD, previous literature reported the MCID for the HADS, triangulated from the distribution-based, anchor-based, and Delphi-based findings, was 1.7 points in cardiovascular diseases (51), meanwhile, the MCID of subscale scores for anxiety and depression were 1.32 and 1.40 in pulmonary diseases, respectively (52).
Secondary outcomes included were: (i) PD-related motor symptoms as measured using the validated Movement Disorder Society-Unified Parkinson's Disease Rating Scale, part III motor examination (MDS-UPDRS-III) (Chinese version) (53), which covers domains related to tremor, rigidity, bradykinesia, gait, and postural instability; (ii) mobility was measured based on the Timed-up-and-go (TUG) test by the United States Food and Drug Administration approved EncephaLog application using a smartphone device (54); (iii) cognition as measured by the Montreal Cognitive Assessment (MoCA) protocol (Hong Kong version) (55), which consists of seven subtests that examine varying cognitive domains, including visuospatial/executive, naming, memory, attention, language, abstraction, and orientation; (iv) mindfulness as measured using the 20-item Five-Facet Mindfulness Questionnaire (Short-form) (FFMQ-SF) (Chinese version), which assesses five mindfulness domains including observing, describing, acting with awareness, nonjudgment of inner experience, and non-reaction to inner experience. FFMQ-SF has been validated in community adults recruiting from out-patient clinics (n=156) and community settings (n=230) which shared similar socio-demographic characteristic as compared

to our study participants (Cronbach's alpha = 0.80-0.83) (56); and (v) HRQOL as assessed using the validated Parkinson's Disease Questionnaire-8 (PDQ-8) (Chinese version) (57,58), which yields a summary index score that captures disease-specific HRQOL in terms mobility, activities of daily living, emotional wellbeing, social support, cognitions, communication, bodily discomfort and stigma.

Dual use research of concern

Policy information about dual use research of concern

Hazards

Could the accidental, deliberate or reckless misuse of agents or technologies generated in the work, or the application of information presented in the manuscript, pose a threat to:

No	Yes	
☒	☐	Public health
☒	☐	National security
☒	☐	Crops and/or livestock
☒	☐	Ecosystems
☒	☐	Any other significant area

Experiments of concern

Does the work involve any of these experiments of concern:

No	Yes	
☒	☐	Demonstrate how to render a vaccine ineffective
☒	☐	Confer resistance to therapeutically useful antibiotics or antiviral agents
☒	☐	Enhance the virulence of a pathogen or render a nonpathogen virulent
☒	☐	Increase transmissibility of a pathogen
☒	☐	Alter the host range of a pathogen
☒	☐	Enable evasion of diagnostic/detection modalities
☒	☐	Enable the weaponization of a biological agent or toxin
☒	☐	Any other potentially harmful combination of experiments and agents

ChIP-seq

Data deposition

☐ Confirm that both raw and final processed data have been deposited in a public database such as GEO.

☐ Confirm that you have deposited or provided access to graph files (e.g. BED files) for the called peaks.

Data access links<br>*May remain private before publication.*	*For "Initial submission" or "Revised version" documents, provide reviewer access links. For your "Final submission" document, provide a link to the deposited data.*
Files in database submission	*Provide a list of all files available in the database submission.*
Genome browser session<br>(e.g. UCSC)	*Provide a link to an anonymized genome browser session for "Initial submission" and "Revised version" documents only, to enable peer review. Write "no longer applicable" for "Final submission" documents.*

Methodology

Replicates	*Describe the experimental replicates, specifying number, type and replicate agreement.*
Sequencing depth	*Describe the sequencing depth for each experiment, providing the total number of reads, uniquely mapped reads, length of reads and whether they were paired- or single-end.*
Antibodies	*Describe the antibodies used for the ChIP-seq experiments; as applicable, provide supplier name, catalog number, clone name, and lot number.*
Peak calling parameters	*Specify the command line program and parameters used for read mapping and peak calling, including the ChIP, control and index files used.*
Data quality	*Describe the methods used to ensure data quality in full detail, including how many peaks are at FDR 5% and above 5-fold enrichment.*

Software | *Describe the software used to collect and analyze the ChIP-seq data. For custom code that has been deposited into a community repository, provide accession details.*

Flow Cytometry

Plots

Confirm that:

☐ The axis labels state the marker and fluorochrome used (e.g. CD4-FITC).

☐ The axis scales are clearly visible. Include numbers along axes only for bottom left plot of group (a 'group' is an analysis of identical markers).

☐ All plots are contour plots with outliers or pseudocolor plots.

☐ A numerical value for number of cells or percentage (with statistics) is provided.

Methodology

Sample preparation | *Describe the sample preparation, detailing the biological source of the cells and any tissue processing steps used.*

Instrument | *Identify the instrument used for data collection, specifying make and model number.*

Software | *Describe the software used to collect and analyze the flow cytometry data. For custom code that has been deposited into a community repository, provide accession details.*

Cell population abundance | *Describe the abundance of the relevant cell populations within post-sort fractions, providing details on the purity of the samples and how it was determined.*

Gating strategy | *Describe the gating strategy used for all relevant experiments, specifying the preliminary FSC/SSC gates of the starting cell population, indicating where boundaries between "positive" and "negative" staining cell populations are defined.*

☐ Tick this box to confirm that a figure exemplifying the gating strategy is provided in the Supplementary Information.

Magnetic resonance imaging

Experimental design

Design type | *Indicate task or resting state; event-related or block design.*

Design specifications | *Specify the number of blocks, trials or experimental units per session and/or subject, and specify the length of each trial or block (if trials are blocked) and interval between trials.*

Behavioral performance measures | *State number and/or type of variables recorded (e.g. correct button press, response time) and what statistics were used to establish that the subjects were performing the task as expected (e.g. mean, range, and/or standard deviation across subjects).*

Acquisition

Imaging type(s) | *Specify: functional, structural, diffusion, perfusion.*

Field strength | *Specify in Tesla*

Sequence & imaging parameters | *Specify the pulse sequence type (gradient echo, spin echo, etc.), imaging type (EPI, spiral, etc.), field of view, matrix size, slice thickness, orientation and TE/TR/flip angle.*

Area of acquisition | *State whether a whole brain scan was used OR define the area of acquisition, describing how the region was determined.*

Diffusion MRI ☐ Used ☐ Not used

Preprocessing

Preprocessing software | *Provide detail on software version and revision number and on specific parameters (model/functions, brain extraction, segmentation, smoothing kernel size, etc.).*

Normalization | *If data were normalized/standardized, describe the approach(es): specify linear or non-linear and define image types used for transformation OR indicate that data were not normalized and explain rationale for lack of normalization.*

Normalization template | *Describe the template used for normalization/transformation, specifying subject space or group standardized space (e.g. original Talairach, MNI305, ICBM152) OR indicate that the data were not normalized.*

Noise and artifact removal | *Describe your procedure(s) for artifact and structured noise removal, specifying motion parameters, tissue signals and physiological signals (heart rate, respiration).*

Volume censoring | *Define your software and/or method and criteria for volume censoring, and state the extent of such censoring.*

Statistical modeling & inference

Model type and settings | *Specify type (mass univariate, multivariate, RSA, predictive, etc.) and describe essential details of the model at the first and second levels (e.g. fixed, random or mixed effects; drift or auto-correlation).*

Effect(s) tested | *Define precise effect in terms of the task or stimulus conditions instead of psychological concepts and indicate whether ANOVA or factorial designs were used.*

Specify type of analysis: ☐ Whole brain ☐ ROI-based ☐ Both

Statistic type for inference (See Eklund et al. 2016) | *Specify voxel-wise or cluster-wise and report all relevant parameters for cluster-wise methods.*

Correction | *Describe the type of correction and how it is obtained for multiple comparisons (e.g. FWE, FDR, permutation or Monte Carlo).*

Models & analysis

n/a	Involved in the study
☐	☐ Functional and/or effective connectivity
☐	☐ Graph analysis
☐	☐ Multivariate modeling or predictive analysis

Functional and/or effective connectivity | *Report the measures of dependence used and the model details (e.g. Pearson correlation, partial correlation, mutual information).*

Graph analysis | *Report the dependent variable and connectivity measure, specifying weighted graph or binarized graph, subject- or group-level, and the global and/or node summaries used (e.g. clustering coefficient, efficiency, etc.).*

Multivariate modeling and predictive analysis | *Specify independent variables, features extraction and dimension reduction, model, training and evaluation metrics.*
