# Supplementary material for: A randomized clinical trial of mindfulness meditation versus exercise in Parkinson’s disease during social unrest
Source: NPJ Parkinsons Dis. 2023 Jan 21;9:7. doi: 10.1038/s41531-023-00452-w (PMC9862216; doi:10.1038/s41531-023-00452-w)
Supplement: Supplementary file 2 — Supplementary table 1; Supplementary table 2; Supplementary table 3 [file 41531_2023_452_MOESM2_ESM.pdf]

Supplementary information

Supplementary table 1. Characteristics of drop-out and non-drop-out cases

Characteristics	Non-drop-out (n=61)	Drop-out (n=7)	χ^2/t	*p*
Age, mean (SD), years[2]	63.9 (8.5)	69.6 (6.3)	1.72	0.090
Female[1]	36 (59.0%)	3 (42.9%)	0.67	0.449
Marital status[1]				
- Single/separated/divorced/widowed	15 (24.6%)	2 (28.6%)	0.053	1.000
- Married	46 (75.4%)	5 (71.4%)		
Body Mass Index[2]	22.7 (3.9)	23.0 (6.2)	0.15	0.884
Education[1]			0.190	1.000
- Illiterate/Primary	13 (21.3%)	1 (14.3%)		
- Secondary or above	48 (78.7%)	6 (85.7%)		
Religion			1.38	0.710
- Christianity	9 (14.8%)	1 (14.3%)		
- Catholicity	5 (8.2%)	1 (14.3%)		
- Buddhism	9 (14.8%)	0 (0%)		
- No religion	38 (62.3%)	5 (71.4%)		
Living status[1]				
- Alone	9 (14.8%)	1 (14.3%)	0.001	1.000
- With spouses/ families/friends	57 (85.2%)	6 (85.7%)		
Social security allowance[1]	31 (50.8%)	5 (71.4%)	1.75	0.416
Disease staging[1]			2.11	0.230
- Mild PD, H&Y stage I or II	26 (42.6%)	1 (14.3%)		
- Moderate PD, H&Y stage III	35 (57.4%)	6 (85.7%)		
Levodopa equivalent dose, mean (SD)[2]	580.5 (565)	1147 (634.9)	2.48	0.016
Charlson Comorbidity Index[2]	0.1 (0.4)	0.1 (0.4)	-0.03	0.979
Schwab and England Scale[2]	9.0 (1.6)	7.1 (3.2)	-1.55	0.170

[1]Categorical and [2]continuous variables were compared between the two groups using the Chi-square test (χ^2) and Independent t-test (*t*), respectively. Data are presented as mean (standard deviation), n (%), or median (interquartile range).

Supplementary information

Supplementary table 2 Summary of process evaluation using satisfaction questionnaire

	Mindfulness meditation (n=31)					Stretching and resistance training exercise (n = 34)				
	Strongly agree	Agree	Neutral	Disagree	Strongly disagree	Strongly agree	Agree	Neutral	Disagree	Strongly disagree
1. The content of the programme is appropriate.	77.4 %	19.4 %	3.2 %	0.0 %	0.0 %	82.4 %	14.7 %	2.9 %	0.0 %	0.0 %
2. The total number of classes and duration of each class was appropriate.	87.1 %	9.7 %	0.0 %	3.2 %	0.0 %	67.6 %	29.4 %	0.0 %	2.9 %	0.0 %
3. The venue was appropriate.	29.0 %	71.0 %	0.0 %	0.0 %	0.0 %	32.4 %	67.6 %	0.0 %	0.0 %	0.0 %
4. I think that the difficulty level of exercise being taught in classes is appropriate for me.	38.7 %	48.4 %	12.9 %	0.0 %	0.0 %	41.2 %	55.9 %	2.9 %	0.0 %	0.0 %
5. I think that the course content is useful and relevant for me.	38.7 %	32.3 %	29.0 %	0.0 %	0.0 %	41.2 %	58.8 %	0.0 %	0.0 %	0.0 %
6. The information being provided related to the programme is appropriate.	51.6 %	45.2 %	0.0 %	0.0 %	0.0 %	38.2 %	58.8 %	2.9 %	0.0 %	0.0 %

7. I am satisfied with the teaching methods delivered by the instructor.	77.4 %	22.6 %	0.0 %	0.0 %	0.0 %	82.4 %	17.6 %	0.0 %	0.0 %	0.0 %
8. I can remember the exercises learned from class.	29.0 %	48.4 %	9.7 %	12.9 %	0.0 %	47.1 %	47.1 %	5.9 %	0.0 %	0.0 %
9. After the completion of programme, I am confident that I could continue the mindfulness/exercise practice in daily living.	38.7 %	45.2 %	9.7 %	6.5 %	0.0 %	47.1 %	52.9 %	0.0 %	0.0 %	0.0 %
10. Overall speaking, I am satisfied with the programme.	64.5 %	32.3 %	3.2 %	0.0 %	0.0 %	73.5 %	26.5 %	0.0 %	0.0 %	0.0 %

Supplementary information

Supplementary table 3. Overview of the modified mindfulness meditation program

Week	**Themes for mindfulness meditation*** (Each session consisted of 15-min introduction, 60-min mindfulness meditation and 15-min closing)	**Homework** (20-min each, twice a week)
1	**Concentration: Body focus**<br>• Mindful breathing<br>• Body scan<br>• Sound bath meditation	• Mindful breathing<br>• Body scan
2	**Concentration: Gathering the scattered mind**<br>• Mindful breathing<br>• Body scan<br>• Sound bath meditation	• Mindful breathing<br>• Body scan
3	**Concentration: Open awareness**<br>• Mindful breathing<br>• Mindful walking<br>• Sound bath meditation	• Mindful walking<br>• Body scan
4	**Clarity: Recognising reactivity** (about detection skills – ability to pick up on subtle or faint sensations)<br>• Raisin exercise<br>• Sitting meditation<br>• Sound bath meditation	• Sitting meditation<br>• Mindfulness in any routine ordinary daily activity (e.g. walking, exercise, sitting, simple physical tasks, etc), focused on detection skills
5	**Clarity: Recognising reactivity** (about discrimination skills – ability to distinguish different types of body sensations and to clearly track where sensations are located in the body)<br>• Mindful breathing<br>• Sitting meditation<br>• Sound bath meditation	• Body scan<br>• Mindfulness in any routine ordinary daily activity, focused on discrimination skills
6	**Equanimity: Allowing and letting be**<br>• Mindful breathing<br>• Body scan<br>• Sound bath meditation	• Body scan<br>• Any kind of formal meditation practice based on participant's preference
7	**Equanimity: Appreciation and gratitude**<br>• Mindful breathing<br>• Loving-kindness meditation<br>• Sound bath meditation	• Loving-kindness meditation<br>• Any kind of formal meditation practice based on participant's preference
8	**Equanimity: Integrating mindfulness for life**<br>• Mindful breathing<br>• Mindful walking<br>• Sound bath meditation	• Any kind of formal meditation practice based on participant's preference<br>• Integrating mindfulness in any routine ordinary daily activity

*Each session started with 15-min of introduction and sharing, follows by 60-min of guided meditation techniques, and 15-min of closing, sharing and briefing of homework. For the first session, the introduction covers three core skills of mindfulness (including concentration, clarity and equanimity), and posture options for meditation practices. For the following sessions, the introduction covered the recap of previous content and sharing of home practice.
